# Intraday seasonalities and nonstationarity of trading volume in financial markets: Collective features

**DOI:** 10.1371/journal.pone.0179198

**Published:** 2017-07-28

**Authors:** Michelle B. Graczyk, Sílvio M. Duarte Queirós

**Affiliations:** 1 Centro Brasileiro de Pesquisas Físicas, Rio de Janeiro, RJ, Brazil; 2 National Institute of Science of Technology for Complex Systems, Rio de Janeiro, RJ, Brazil; University of Rijeka, CROATIA

## Abstract

Employing Random Matrix Theory and Principal Component Analysis techniques, we enlarge our work on the individual and cross-sectional intraday statistical properties of trading volume in financial markets to the study of collective intraday features of that financial observable. Our data consist of the trading volume of the Dow Jones Industrial Average Index components spanning the years between 2003 and 2014. Computing the intraday time dependent correlation matrices and their spectrum of eigenvalues, we show there is a mode ruling the collective behaviour of the trading volume of these stocks whereas the remaining eigenvalues are within the bounds established by random matrix theory, except the second largest eigenvalue which is robustly above the upper bound limit at the opening and slightly above it during the morning-afternoon transition. Taking into account that for price fluctuations it was reported the existence of at least seven significant eigenvalues—and that its autocorrelation function is close to white noise for highly liquid stocks whereas for the trading volume it lasts significantly for more than 2 hours —, our finding goes against any expectation based on those features, even when we take into account the Epps effect. In addition, the weight of the trading volume collective mode is intraday dependent; its value increases as the trading session advances with its eigenversor approaching the uniform vector as well, which corresponds to a soar in the behavioural homogeneity. With respect to the nonstationarity of the collective features of the trading volume we observe that after the financial crisis of 2008 the coherence function shows the emergence of an upset profile with large fluctuations from that year on, a property that concurs with the modification of the average trading volume profile we noted in our previous individual analysis.

## Introduction

The relevance of the trading volume in stock trading is more than well-established: the decision of buying and selling is mainly prompted by factors that bidders and askers believe to affect the price *S* and its reckoning that the stock is underpriced(overpriced). In other words, setting particular episodes apart—*e.g*., dividend or interest payments, (reverse) stock splits –, the price changes when a buyer and a seller agree on making a transaction at some new price *S*. In this context, the transfer of equity between agents—the trading volume—can act as a proxy for the flow of information among them. Large trading volumes are immediately associated with a lot of ‘buzz’ which tend to reflect on the price [1].

In the scenario where the trading volume is a proxy for market information, we can study the relations between trading volume, price fluctuations and information. The first approach blending these three quantities is the Mixture of Distributions Hypothesis (MDH) introduced by Clark [2] which conjectures that, since the dynamics of the volatility and the trading volume are dependent on latent events (disclosure of information), there is a joint distribution for these two quantities with both quantities marginally follow a log-Normal distribution. Additionally, Clark defined a difference between physical (clock) and proper (event) time at which information is input, a difference of concepts that also turns up being fundamental in the description of complex systems and critical phenomena [3]. As examples of a quantitative approaches assuming the MDH, we point to studies on the stochastic nature of the volatility [4–7] as well as ARCH-like heteroskedastic models [8], which consider the price fluctuations and trading volume share the same underlying [9].

Contrasting with the MDH synchronous nature of the trading volume and volatility dynamics, Copeland introduced the Sequential Arrival of Information Hypothesis (SAIH) [10] which asserts information reaches the trading agents at different times so that the final state of the market is attained by a sequence of local stationary states. In that case, there is a delay in the maximal correlation between volatility and the trading volume.

In a previous paper of ours, hereinafter referred to as Paper I [11], we introduced a broad study over the individual and cross-sectional intraday statistical properties of the trading volume of blue chip equities that composed the Dow Jones Industrial Average with the aiming of going beyond the well-known intraday ∪-shape of the average trading volume, a profile that is also shared by different definitions of the volatility, including the absolute value of the price fluctuations [1, 12–16]. Paper I showed important features of the trading volume such as the fact the morning (am) and the afternoon (pm) parts of the business day clearly have different dynamical mechanisms of trading. Therein, we related that property to an important change in the way the market participants transfer information between them during the two parts of the session; furthermore, we concluded that such difference is now significantly larger than before the 2008 crisis. That evolution has been accompanied by an overall reduction of the concavity of the ∪-shape. In addition, we computed a ∽-shape for the kurtosis in both the individual and the cross-sectional analyses, with the former being always greater than 3 (leptokurtic) whereas the latter approaches 3 (Gaussianity) by the middle of the morning and by the end of the session. The message we extracted from combining all those statistical properties is that the higher level of trading in the morning is related to equities whose overnight news must be transferred to their prices whereas the increase in the trading activity that is observed in the last part of the session corresponds to an overall augment in the level of activity, which is largely fuelled by the need for closing positions. These results add to other established properties of the trading volume [16] such as the asymptotic power-law distribution [17–20], long-lasting correlations [20, 21] and (multi-)scaling [22, 23]. Besides the statistical description of the trading volume, related quantities such as the trading value [24–28]—the product of the price by the trading volume—and the trading volume fluctuations [29] has been studied and provided important insights into financial dynamics.

Although the cross-sectional analysis already furnished some information on the mutual behaviour of the trading volume, we can obtain a further and more detailed insight into its collective features utilising Principal Component Analysis (PCA) and Random Matrix Theory (RMT). The latter was first applied by E.P. Wigner to explain the energy levels of atomic nuclei [30–32]. The idea that the complex nature of their constituents and the interactions between them would be best described by stochastic elements has found resemblance in a wide range of physical [30, 33–36] and non-physical phenomena [37–40], including Quantitative Finance [41–50], namely in the collective analysis of price fluctuations for the purposes of portfolio management and risk assessment.

With this paper, we seek to understand how the trading volume collectively behaves across the trading session from a random matrix perspective. To that, we follow along the lines presented in the section ‘Materials and Methods’; in the section ‘Results’ we first analyse the general collective properties of the trading volume in a financial market and in a second stage and study how these properties have evolved in recent years. Finally, in ‘Conclusion’ we address the impact of our results on trading modelling and set forth prospective issues worth studying.

## Materials and methods

### Materials

Our results are obtained from the same data of Paper I: 1-minute trading volume spanning the period between the 4th January 2004 and the 30th December 2013 of the 30 companies composing the Dow Jones Industrial Average. These data have been provided by Olsen Financial Data and the Chair of Quantitative Finance of the École CentraleSupélec. (The former has furnished the data for the second semester of 2004 which we used for a first analysis and the former has supplied data corresponding to the entire set from which the full results are obtained.) The companies and their corresponding tickers are listed in Table 1, where the suffix ‘.N’ signals the equity is traded at NYSE and ‘.OQ’ at NASDAQ, respectively. Both markets—regardless of (pre)post-market periods—open at 9:30 and close at 16:00. We employ the notation *v*_*i*_(*t*, *d*; *s*) to represent the trading volume of company *i* at time *t* and day *d*. The former is shown in the form of an integer number so that 9:30 → *t* = 1, …, 16:00 → *t* = 391. On top of that, we also take into account the semester, *s*, day *d* belongs to. We do so because we divide our data into semesters in order to assess the nonstationarity of the intraday properties. Partitioning our data that way yields a good balance between quasi-stationarity and the number of days within each interval so that a significant analysis can be carried out. Such segmenting is performed twofold, overlapping the semesters and nonoverlapping them. In the overlapping semester scheme the 6-month period comprising the months from January to June of 2004 corresponds to *s* = 1, from February to July of 2004 is *s* = 2, from March to August of 2004 is denoted by *s* = 3 and so forth whereas in the nonoverlapping contiguous approach we make use of the notation xSyy where x = {1, 2} represents the first(second)—i.e., from January(July) to June(December)—of the year 20yy.

**Table 1:** Companies analysed and their ticker symbols.

Company	Ticker symbol
Alcoa Inc.	AA.N
American International Group Inc.	AIG.N
American Express Co.	AXP.N
Boeing Co.	BA
Citigroup Inc.	C.N
Catterpilar Inc.	CAT.N
E.I. DuPont de Nemours & Co.	DD.N
Walt Disney Co.	DIS.N
General Eletric Co.	GE.N
General Motors Corp.	GM.N
Home Depot Inc.	HD.N
Honeywell International Inc.	HO.N
HewlettPackard Co.	HPQ.N
International Business Machines Corp.	IBM.N
Intel Corp.	INTC.OQ
Johnson & Johnson	JNJ.N
JPMorgan Case & Co.	JPM.N
Coca-Cola Co.	KO.N
McDonald’s Corp.	MCD.N
3M Co.	MMM.N
Altria Group Inc.	MO.N
Merc & Co.	MRK.N
Microsoft Corp.	MSFT.OQ
Pfizer Inc.	PFE.N
Procter & Gamble Co.	PG.N
SBC Communications Inc. -> AT&T Inc.	SBC.N -> T.N
United Technologies Corp.	UTX.N
Verizon Communications Inc.	VZ.N
Wal-Mart Stores Inc.	WMT.N
Exxon Mobil Corp.	XOM.N

### Methods

#### *F*-test on the equality of the variances

Sets of random variables can be different in a multitude of ways, eg, in respect of the average, the variance or generically the distribution itself. When we want to compare the variances of (sub)sets of random variables the *F*- test on the equality of the variances is assumed as the standard test. In general, and for two populations, that test corresponds to the computation of the ratio,

$$\begin{array}{c} F=\frac{\sigma_{x}^{2}}{\sigma_{y}^{2}} \end{array}$$ (1)

where

$$\begin{array}{c} \sigma_{\left(.\right)}^{2}=\frac{1}{n_{\left(.\right)}-1}\;\sum_{l=1}^{n_{\left(.\right)}}\;{\left(x_{l}-\bar{x}\right)}^{2} \end{array}$$ (2)

corresponding to the variances of two independent and identically distributed sets of random variables {*x*} and {*y*} with *n*_*x*_ and *n*_*y*_ elements following distributions whose averages are equal to $\mu_{x}=\frac{1}{n_{x}}\;\sum_{l=1}^{n_{x}}\;x_{l}$ and $\mu_{y}=\frac{1}{n_{y}}\;\sum_{l=1}^{n_{y}}\;y_{l}$, respectively. The variable *F* is associated with an *F*-distribution,

$$\begin{array}{c} p\left(F\right)\propto F^{\frac{n_{x}}{2}-1}\;{\left(1+\frac{n_{x}}{n_{y}}F\right)}^{-\frac{n_{x}+n_{y}}{2}}. \end{array}$$ (3)

Testing the equality of the variances the null hypothesis assumes the ratio Eq (1) must be equal to 1, ie, the variances are equal. In practice, the value of *F* must be compared to

$$\begin{array}{c} F^{stat}\equiv \frac{\text{explained}\;\text{variance}}{\text{unexplained}\;\text{variance}}, \end{array}$$ (4)

where the numerator reads,

$$\begin{array}{c} \sum_{u=\{x,y\}}n_{u}\;{\left(\mu_{u}-\mu^{2}\right)}^{2} \end{array}$$ (5)

and the denominator

$$\begin{array}{c} \sum_{u=\{x,y\}}\;\sum_{l=1}^{n_{u}}n_{u}\;{\left(X_{lu}-\mu_{u}\right)}^{2}, \end{array}$$ (6)

({*X*} ≡ {*x*} ∪ {*y*}). If—at a significance level *α*—$F_{\alpha /2,n_{x}-1,n_{y}-1}^{stat}$ is smaller than *F* value, or else $F_{1-\alpha /2,n_{x}-1,n_{y}-1}^{stat}$ is bigger than the *F* value, where $F_{\alpha ,n_{x}-1,n_{y}-1}^{stat}$ is the critical value of the *F*-distribution with *n*_*x*_ − 1 and *n*_*y*_ − 1 degrees of freedom and a significance level of *α*, then we can reject the null hypothesis.

#### Fundamentals of random matrix theory in a nutshell

For every company *i*, we can group the trading volume *v*_*i*_(*t*, *d*; *s*) into a set constrained to the parameter *d* (and thus to *s* as well), {*v*_*i*_(*t*, *d*; *s*)|_*d*, *s*_} with *N*_*D*_ elements corresponding to the number of days in semester *s* with active trading at time *t*. The combination of these *N* × *N*_*D*_ random variables is used to define the *N* × *N* correlation matrix, C (*t*; *s*) whose entries correspond to the Pearson’s correlation coefficient,

$$\begin{array}{c} C\;{\left(t;s\right)}_{ij}\equiv \frac{\bar{v_{i}\left(t,d;s\right)\;v_{j}\left(t,d;s\right)}-\mu_{i}\left(t;s\right)\;\mu_{j}\left(t;s\right)}{\sigma_{i}\left(t;s\right)\;\sigma_{j}\left(t;s\right)}, \end{array}$$ (7)

(−1 ≤ C (*t*; *s*)_*ij*_ ≤ 1), where the overline represents that we have averaged over the number *N*_*D*_ of valid days in that semester. The average, *μ*_*i*_(*t*; *s*), and the standard deviation, *σ*_*i*_(*t*; *s*), are obtained by carrying out the statistics over *N*_*D*_ as well. According to Eq (7), the elements of the diagonal of the correlation matrix are equal to one, and therefore the trace of C (*t*; *s*) is always equal to 30.

Taking into consideration the RMT, we can identify important properties of the correlation matrices Eq (7); one of them corresponds to the probabilistics of the spectrum of eigenvalues {λ}. In asymptotic the limit *N*, *N*_*D*_ → ∞ with $1<\frac{N_{D}}{N}<\infty$, that spectrum follows the Marchenko-Pastur distribution:

$$\begin{array}{c} \rho_{t;s}\left(\lambda \right)=\frac{N_{D}}{N}\frac{\sqrt{\left(\lambda_{+}-\lambda \right)\left(\lambda -\lambda_{-}\right)}}{2\pi \lambda }. \end{array}$$ (8)

for λ_−_ ≤ λ ≤ λ_+_ and *ρ*_*t*;*s*_(λ) = 0, otherwise. The symbols λ_±_ represent the maximal(minimal) eigenvalue and they read

$$\begin{array}{c} \lambda_{\pm }=1+\frac{N}{N_{D}}\pm 2\sqrt{\frac{N}{N_{D}}} \end{array}$$ (9)

The eigenvalues larger than the maximal eigenvalue λ_+_ establish the meaningful structure of the correlations of the multivariate stochastic variable whereas the remaining eigenvalues lying within the interval [λ_−_, λ_+_]—the Marchenko-Pastur ‘sea’—are equivalent to random noise. Accordingly, the correlation matrix corresponds to the superposition of two other correlation matrices,

$$\begin{array}{c} C\;(t;s)=C_{\text{rnd}}\;(t;s)+C_{\text{str}}\;(t;s) \end{array}$$ (10)

where the random part, C_rnd_ (*t*; *s*), is composed of the eigenvalues within the ‘sea’ whereas as the structural counterpart, C_str_ (*t*; *s*) consists of the eigenvalues larger than λ_+_.

If the largest observed eigenvalue λ_max_ is larger than maximum eigenvalue λ_+_ and (much) larger than the others eigenvalues and its corresponding eigenvector, ${\vec{v}}_{\max}$, has all of its components greater than zero, we can associated these eigencomponents with a market mode.

#### Spectral Decomposition of a correlation matrix

In practical terms, the finiteness of the data affects the determination of the correlation matrix entries, Eq (7), which might reflect on unrealistic results such as the computation of negative eigenvalues. In order to filter specious contributions to the correlation matrix, we apply the method known as Spectral Decomposition within the Analysis of Principal Component [50].

If S is the self-system of a real and symmetric matrix U and {λ_*i*_} the set of the eigenvalues of U, then the following identity

$$\begin{array}{c} U.S=\Lambda .S,\;(\Lambda =\text{diag}\;(\lambda_{i})) \end{array}$$ (11)

holds. Moreover, we can define the nonzero elements of the diagonal matrix Λ′ as

$$\begin{array}{ccc} \Lambda^{\prime } & : & \lambda_{i}^{\prime }=\{\begin{array}{c} \begin{array}{cc} \lambda_{i}: & \lambda_{i}\geq 0\\ 0: & \lambda_{i}<0 \end{array} \end{array} \end{array}$$ (12)

We can also defined the non-zero elements of the scaling matrix T from the self-system S by:

$$T\;:\;t_{i}={\left[\sum_{m}\;s_{im}^{2}\lambda_{m}^{\prime }\right]}^{-1}$$ (13)

Taking

$$\begin{array}{c} B^{\prime }=S\;\sqrt{\Lambda^{\prime }} \end{array}$$ (14)

and

$$\begin{array}{c} B=\sqrt{T}\;B^{\prime }=\sqrt{T}\;S\;\sqrt{\Lambda^{\prime }} \end{array}$$ (15)

we have

$$\begin{array}{c} \hat{U}=B\;B^{T} \end{array}$$ (16)

where $\hat{U}$ is positive semi-definite and have all the elements of the main diagonal equal to 1.

That being said, we can resume the method as follows:

1. compute the eigenvalues λ_*i*_ of U and their eigenvectors *s*_*i*_;
2. assume all the negative eigenvalues λ_*i*_ as null;
3. Multiply each eigenvector *s*_*i*_ by its adjusted associated eigenvalue $\lambda_{i}^{\prime }$ to get the columns of B′ and;
4. Obtain B from the normalization of the vectors lines of B′.

## Results and discussion

### Intraday dynamics

#### Eigenvalue analysis

**Fig 1:**
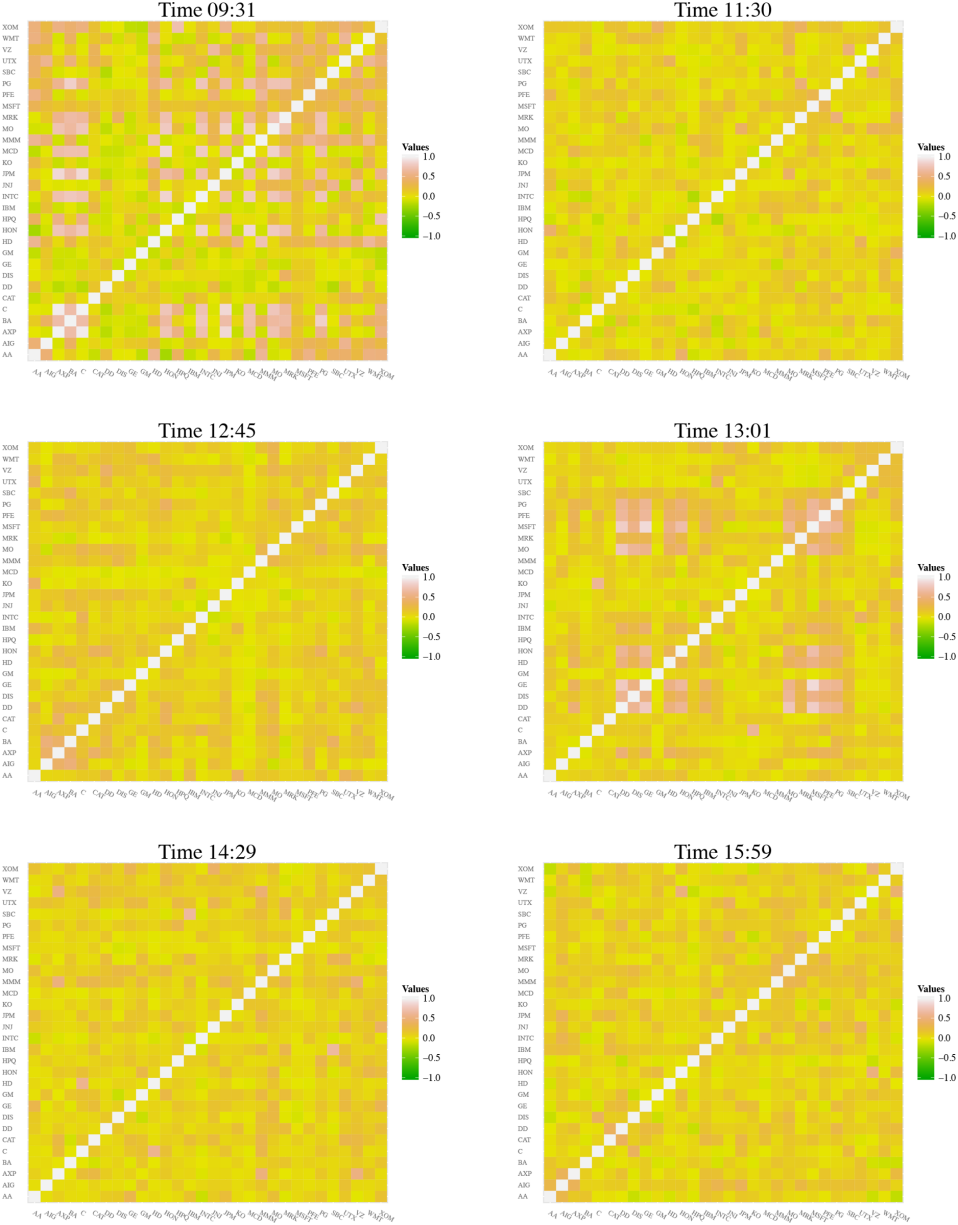
Correlation matrix C(*t*; 2*S*04) of the trading volume of the DJIA companies for the times indicated in the title of each panel.

We start our analysis on the collective behaviour of the trading volume by computing the eigenvalues and the eigenvectors of the correlation matrix C(*t*; *s*) whose entries are defined by Eq (7). The typical evolution of that matrix is shown in Fig 1 for some illustrative timestamps and for the entire trading session in the video available in S1 Video for 2*S*04. The analysis of those plots allows us to grasp that there are specific times, namely the opening of the market (*t* ≈ 1) and the (effective) transition between the morning and the afternoon (timestamp *t* ≈ 190) where the correlations are stronger on average. Specifically, we observe that browner(greener) regions emerge at those times.

**Fig 2:**
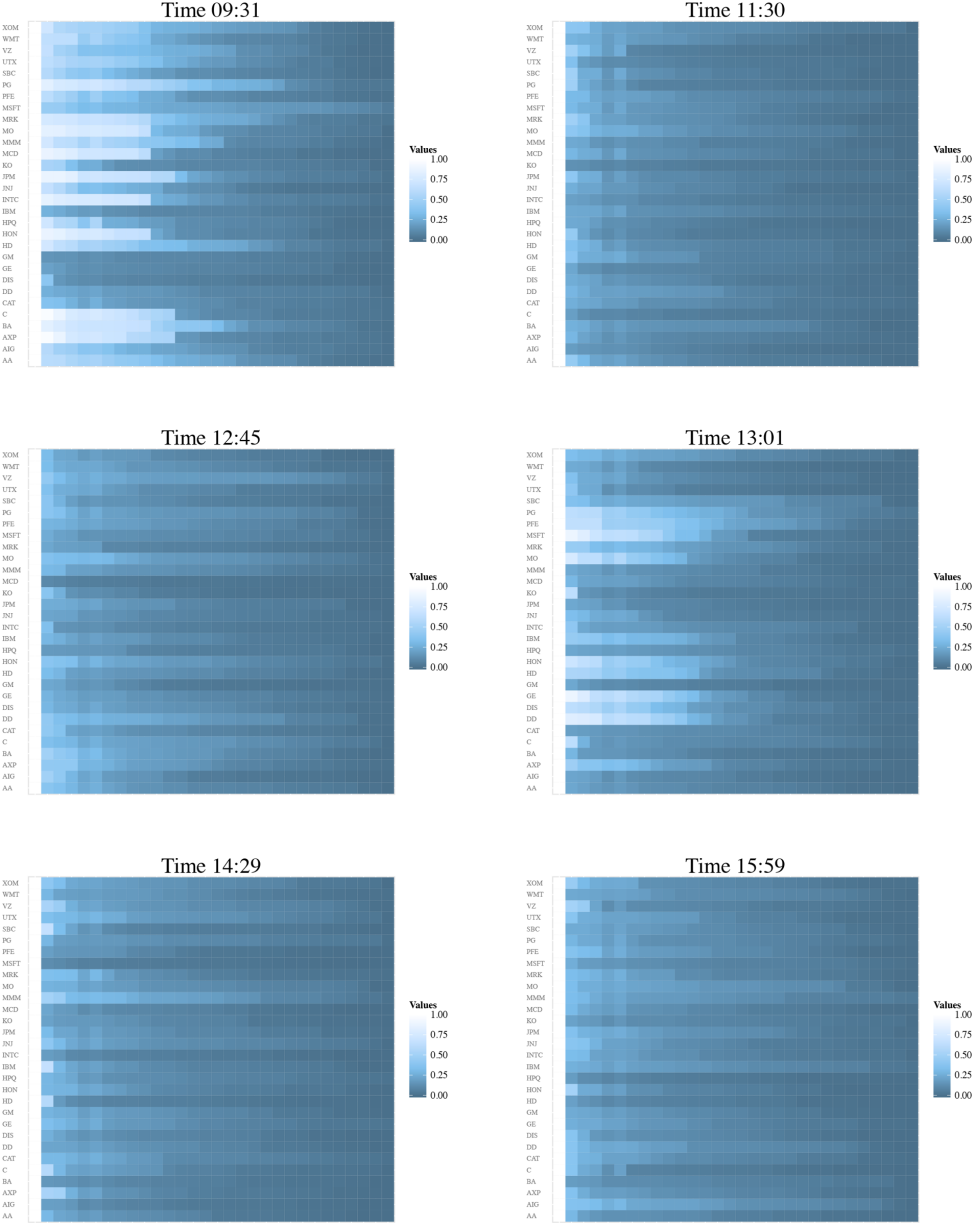
Sorted out absolute correlation matrix C(*t*; 2*S*04) (in descending order along the lines) of the trading volume of the DJIA companies for the times indicated in the title of each panel. N.B.: Because of the ordering process we have applied to get the matrices C_s_(*t*; *d*), they loose the properties of a true correlation matrix, namely that for normalised variables Tr C_s_(*t*; *d*) ≠ *N*, where *N* defines the number of rows and columns of the matrix.

That perception is confirmed when we define a matrix, C(*t*; *s*), obtained by considering the absolute values of the entries of C(*t*; *s*) which are then sorted out—eg, in descending order—along each line, (see Fig 2 and S2 Video for *s* = 2*S*04). In that case, the lighter the region in the plot, the stronger the correlations.

**Fig 3:**
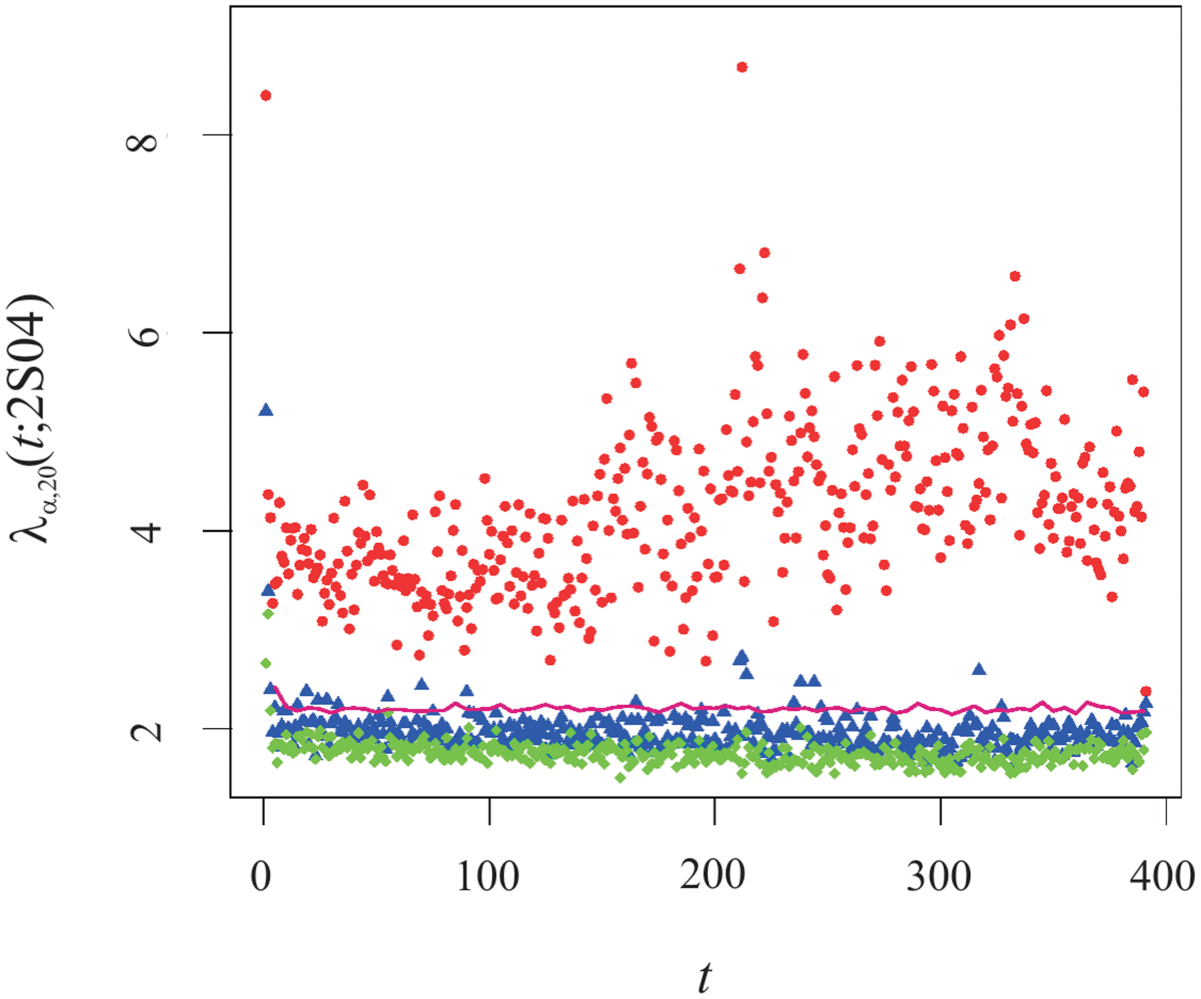
Evolution of the three largest eigenvalues λ_*α* = {1, 2, 3}_(*t*; 2*S*04) of the respective correlation matrix C(*t*; 2*S*04) v intraday time. The legend is the following: λ_1_(*t*; 2*S*04)—red dots, λ_2_(*t*; 2*S*04)—blue triangles, λ_3_(*t*; 2*S*04)—green diamonds and the pink line corresponds to the 95%-level of largest eigenvalue of the correlation matrix computed obtained for *N* independent Gamma distributions with the same mean and variance of DJIA stocks trading volume at time *t*.

From a purely quantitative perspective, the main analysis that can be carried out on a (random) matrix is the determination of its spectrum of eigenvalues, {λ_*α*_}, and the set of their associated eigenvectors, $\left\{{\vec{v}}_{\alpha }\right\}$. In Fig 3, we present the intraday profile of the three largest eigenvalues for 2S04. For the largest eigenvalue, λ_1_(*t*; 2*S*04), it is perceivable a 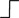-like shape. Applying the *t*-Student test (the details of this test can be found, eg, in the section ‘Materials and Methods’ of Paper I) on the equality of the means, we have *t*-value equal to 12.09 which is much larger than the critical *t*-values that equals 1.654. That implies the two parts of the session are robustly different one another with a significance of 95%. For the case of λ_2_(*t*; *s*) and λ_3_(*t*; *s*), we are unable to identify any relevant difference between the morning and the afternoon parts of the session.

**Fig 4:**
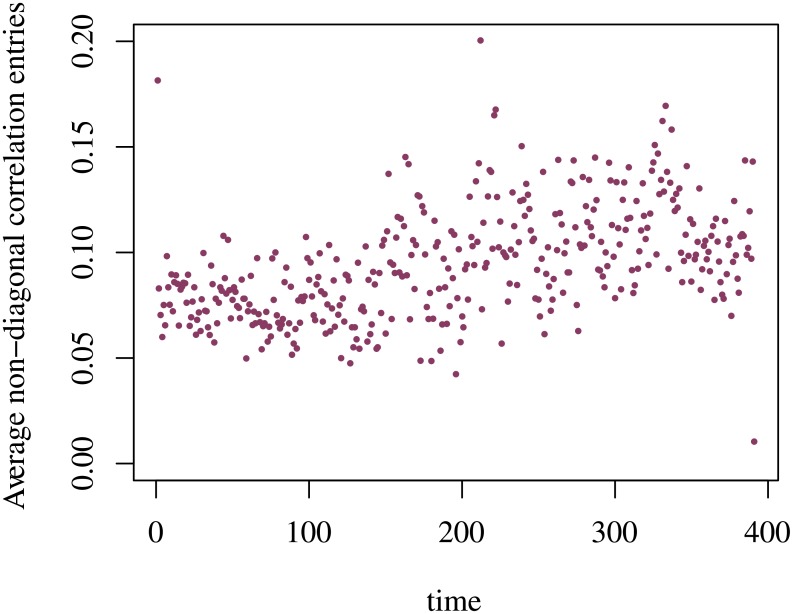
Average value of the non-diagonal elements of the correlation matrix C(*t*; 2*S*04) v intraday time.

Following the theory of random matrices, we can determine the upper and lower bounds, λ_±_, of the Marchenko-Pastur distribution (MPD), Eq (8). For the illustrative semester, 2S04, using Eq (9) we have λ_+_ = 2.2 and λ_−_ = 0.27. Across the trading session, the average value of the largest eigenvalue is equal to 4.2 which is sustainably above λ_+_. From these figures, we confirm that λ_1_ corresponds to a mode of the trading volume, a feature that extends to all the semesters composing our dataset. The identification of that market mode can be further tightened by bringing into effect the rank one perturbation approach onto the correlation matrix; in other words, we assume that C(*t*; *s*) equals the superposition of the Identity Matriz, I, and a zero-diagonal matrix whose (non-diagonal) elements C′(*t*; *s*) have an average value equal to *c*(*t*; *s*). The evolution of *c*(*t*; 2*S*04) is we depicted in Fig 4. Computing Λ = *N* × *c*(*t*; *s*), we verify that such value is not clearly larger than 1, except at the opening and during the morning-afternoon transition. Because the inequality $\Lambda >1+\sqrt{N/T}$ is verified, we are within the same conditions as described in [43, 51] where it is shown that the minimal value for considering an eigenvalue outside the Marchenko-Pastur domain is given by

$$\begin{array}{c} \lambda^{*}=\Lambda +\frac{\Lambda \;\sqrt{N/T}}{\Lambda -1} \end{array}$$ (17)

that yields $\lambda_{\text{am}}^{*}=3.03$ and $\lambda_{\text{pm}}^{*}=4.0$, which are smaller than the average values that equal λ_1,am_ = 3.8 and λ_1,pm_ = 4.6 obtained directly from the data. These values correspond to 12.6% and 16% of Tr C(*t*; *s*) = 30, respectively.

Still, we can check the likelihood of the trading volume market mode by establishing a custom-made statistical test which heeds that the *trading volume is non-negative defined*. The test runs as follows: owing to the previous results on the local statistics of these data, namely that the local distribution of trading volume is well-described by a Gamma distribution [52], for a intraday time *t*, we generate *N* independent Gamma distributed series—with the same number of elements as the number of business days in semester *s*—whose mean and variance match with the values obtained from the data at *t*. From these series, we compute the independent correlation matrix, C_ind_(*t*; *s*) whence a spectrum of eigenvalues is obtained. For each time step, the process we have just described is carried out a large number of times. At each iteration, the value of λ_1_ is stored up. For a large number of samples bearing a large set of λ_1_ values, we sort that set and at the end we pick the last value in the top 5%. Accordingly, that figure establishes the critical value of the statistical significance of obtaining a largest eigenvalue equal to λ_1_(*t*; *s*) associated with a set of independently Gamma distributed variables. The results of that process are depicted by the pink line in Fig 3, from which it is visible that the dots representing λ_1_(*t*; *s*) are steadily and in large measure above that line. This upholds the existence of a trading volume collective mode. On the other hand, looking at the second and third largest eigenvalues we verify they are definitely within the Marchenko-Pastur range, except for the very first minutes of the trading session—where the values of the second largest eigenvalue are robustly above the critical value—and during the morming-afternoon transition in which λ_2_ is a little above it. For the rest of the time, that eigenvalue is approximately 10% below the computed mode limit.

We shall now compare our results with those obtained for the price fluctuations [15]. Explicitly, it was found the collective dynamics presents a strong market mode, with λ_1_/*N* larger than the value we have obtained for the trading volume. The intraday profile of the largest eigenvalue is also marked by a shift upwards in the beginning of the afternoon part of the session; however, in the case of the price fluctuations, the spike in the opening and during the morning-afternoon transition cannot be perceived. Nonetheless, a major difference emerges in respect of the limits of the MPD; we have seen that for the trading volume, the second largest eigenvalue is already close to the upper bound of the MPD distribution for all the semesters we analyse, a feature that is at odds with our expectations. The startlingness over this result stems from the fact that for 5-minute price fluctuations—which are quite close to white-noise for blue chip equities—the authors of [15] have found that the first seven larger eigenvalues surpass λ_+_. We recall that the autocorrelation of the trading volume lasts much longer than that of the price fluctuations, typically two hours for 1-minute trading volume [53, 54]. Besides the fact we are coping with a different financial quantity, we must take into attention that our sample rating is higher than 5-minute frequency used in [15]. In a previous work [55], it was verified that the lag, Δ, assumed in the computation of the price fluctuations, *r*_Δ_(*t*) ≡ ln *S*(*t*) − ln *S*(*t* − Δ), is pivotal in the results of the analysis of the correlation matrix, namely pointing that up to Δ = 5 minutes the correlation structure is in a transient state that arises a manifestation of the Epps effect caused by the asynchronous trading of the companies [56]. In order to clarify whether the collective dynamics of the trading volume of the DJIA companies is affected by asynchrony in trading as well, we analyse the correlation spectrum of the cumulative trading volume

$$\begin{array}{c} V_{i}\left(t,d;\Delta ,s\right)\equiv \sum_{\tau =0}^{\Delta -1}\;v_{i}\left(t-\tau ,d;s\right). \end{array}$$ (18)

**Fig 5:**
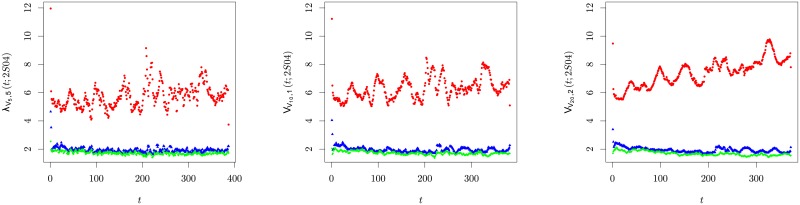
Evolution of the three largest eigenvalues λ_*α* = {1, 2, 3}_(*t*; 2*S*04) of the respective cumulative trading volume, *V*_*i*_(*t*, *d*; Δ, *s*), correlation matrix C_Δ_(*t*; 2*S*04) v intraday time for Δ = 5 minutes (left panel), Δ = 10 minutes (central panel) and Δ = 20 minutes (right panel). The legend is the following: λ_1_(*t*; 2*S*04)—red dots, λ_2_(*t*; 2*S*04)—blue triangles, λ_3_(*t*; 2*S*04)—green diamonds.

In Fig 5, we show the intraday profile of the three largest eigenvalues of C_Δ_(*t*; 2*S*04) with Δ = {5, 10, 20} minutes. Despite the fact the panels in that figure show the trading volume collective mode gets larger as the lag increases, the remaining eigenvalues stay within the Marchenko-Pastur domain (once again apart from the very first values of λ_2_). This result points that asynchrony also enhances the weight of the largest eigenvalue of the trading volume correlation function, but it does not affect the value of the subsequent orders of the eigenspectrum, namely of the second and third largest eigenvalues, which were found significant for the collective behaviour of the price fluctuations at that time scale. (NB: Taking into consideration that Tr C_Δ_ = *N* (for all Δ), an analysis of eigenvalue spectrum shows the augment of λ_1_ is set off by a decrease of the values of the (central) second third, (λ_11_, λ_20_), of the spectrum. The bottom third of the spectrum—namely λ_30_ does not change beyond standard fluctuation.)

#### Eigenversor analysis

The increasing of λ_1_(*t*; *s*) across the business day indicates the equities tend to get more concerted in the afternoon than in the morning. How does this property influence the possible intraday dynamics of the eigenversors, ${\vec{v}}_{\alpha }\left(t\right)$? Focussing on *α* = 1, the structure of each eigenversor—ie, the magnitude of its components—establishes the weight of each equity in the trading volume mode. Moreover, each company can be understood as describing a direction ${\hat{z}}_{i}$ in the DJIA *N*-dimensional trading volume space. That said, the most straightforward way to assess the evolution of the relative weight of each company in the trading volume collective behaviour is to compare the eigenversor related to the largest eigenvalue, with the uniform vector,

$$\begin{array}{c} \vec{u}\equiv \frac{1}{\sqrt{3}0}\;({\hat{z}}_{\text{AA}}+{\hat{z}}_{\text{AIG}}+\ldots +{\hat{z}}_{\text{WMT}}+{\hat{z}}_{\text{XOM}}), \end{array}$$ (19)

computing the scalar product,

$$\begin{array}{c} D\left(t;s\right)\equiv {\vec{v}}_{1}\left(t;s\right)\cdot \vec{u}. \end{array}$$ (20)

**Fig 6:**
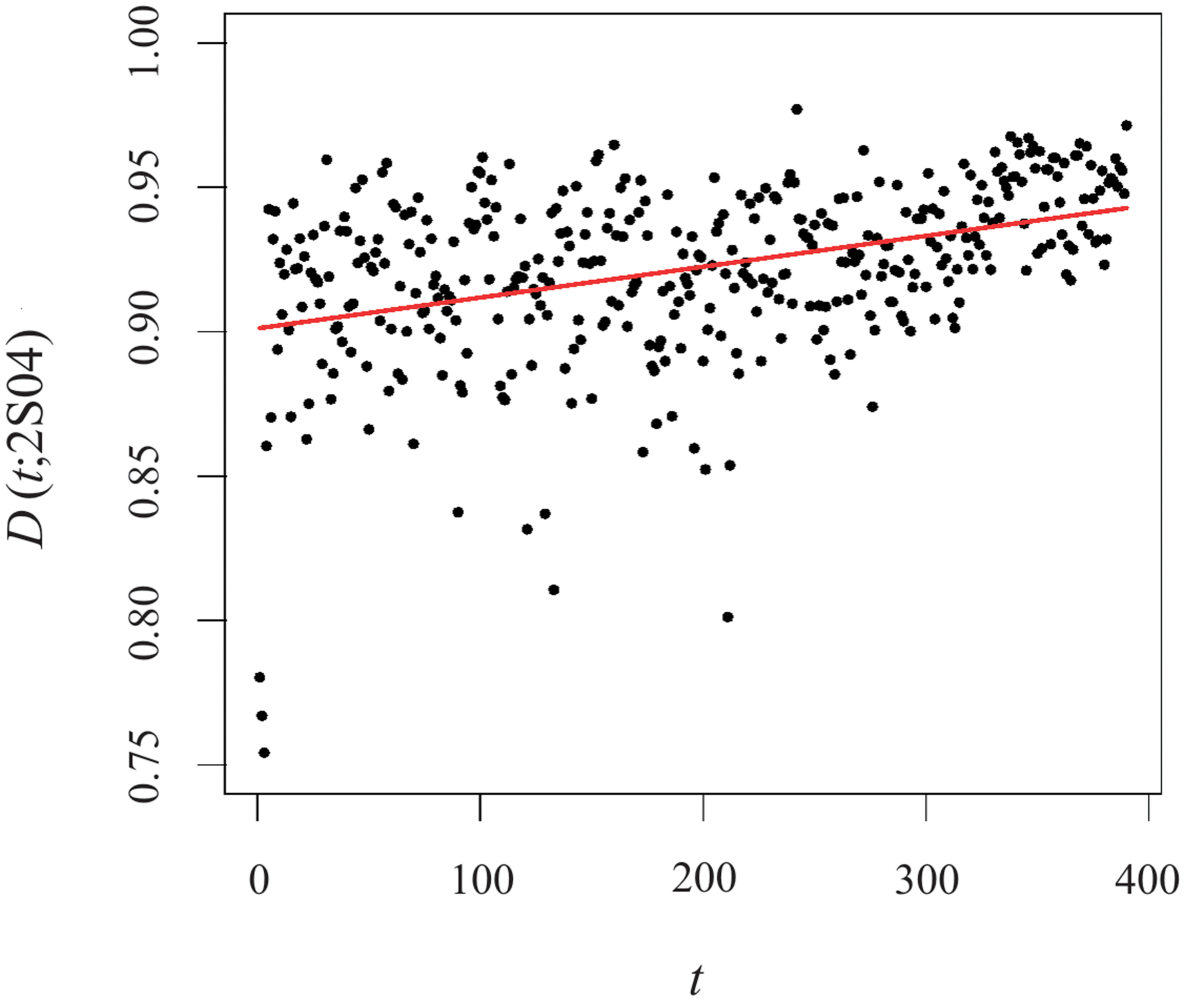
Evolution *D*_1_(*t*; 2*S*04) v intraday time. The value of the slope of the linear fit (solid red line) are reproduced on the plot. The solid grey line at time *t* corresponds to the local arithmetic average over a sample window starting at *t* − 5 and ending *t* + 5.

**Fig 7:**
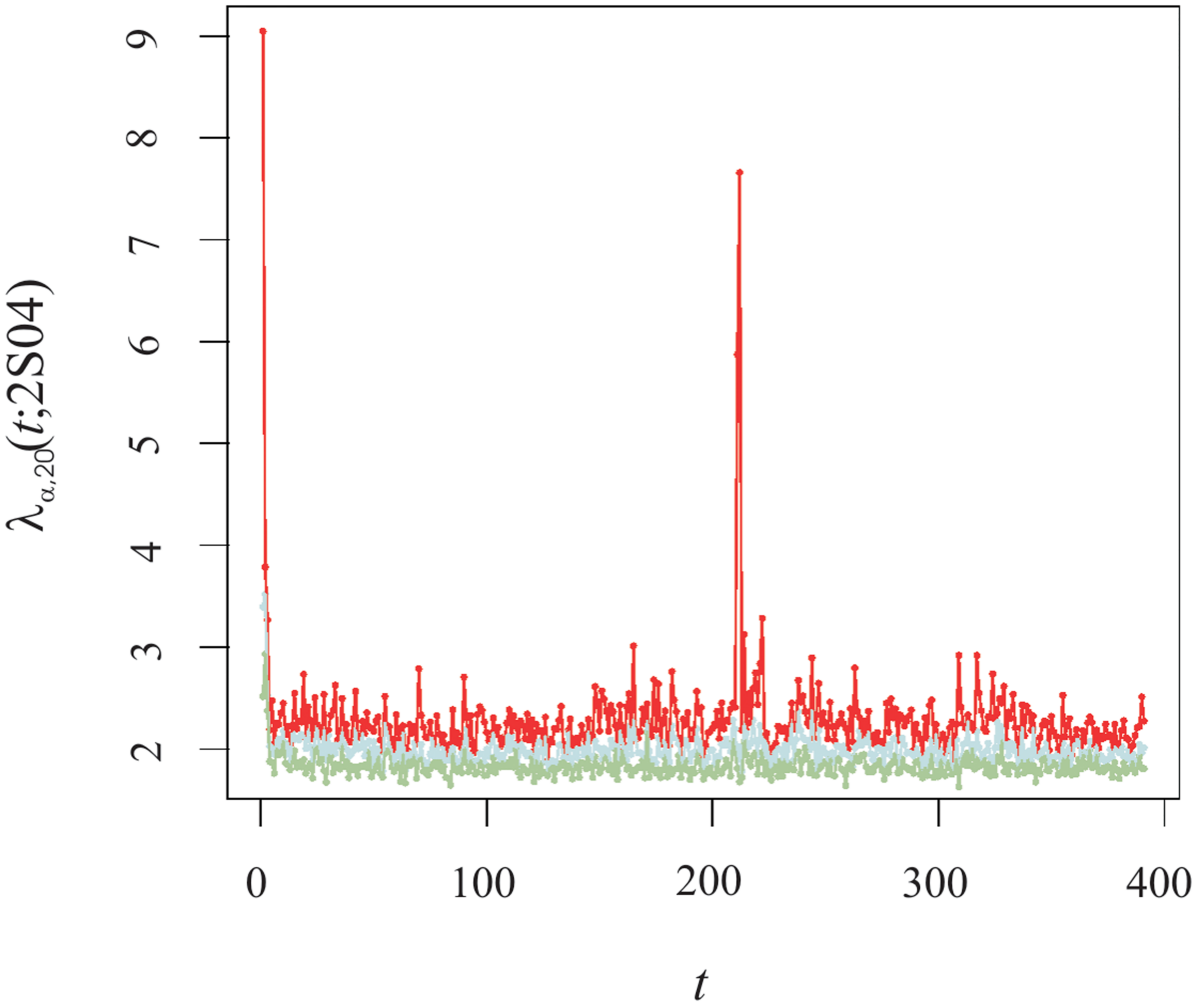
Evolution of the three largest eigenvalues λ_*α*={1,2,3},40_(*t*; 2*S*04) of the respective constrained correlation matrix C_40_(*t*; 2*S*04) v intraday time. The legend goes as follows: λ_1,40_(*t*; 2*S*04)—red, λ_2,40_(*t*; 2*S*04)—blue, λ_3,40_(*t*; 2*S*04)—green.

We note that, although ${\vec{v}}_{1}\left(t;s\right)$ is close to uniformity, the scalar product tends to 1 as the trading session elapses (see Fig 6 and the corresponding first largest eigenvalues are presented in Fig 7). Fitting the results of *D*(*t*; *s*) with a straight line we find a slope around 10^−4^, which is significant regarding its error though. In Fig 6, the smaller (than 0.8) values of *D*(*t*; 2*S*04) are computed for the very first minutes of the session as well as for the morning-afternoon transition. It is worth bridging such results—that indicate a preponderance of some companies with respect to the others—with the augment of the cross-sectional kurtosis of the trading volume reported in Paper I for the same intraday times (a detailed analysis on this matter is addressed in the Conclusion).

#### Constrained correlation analysis

Up to now, we have provided a general account over the trading volume correlations of DJIA stocks. Similarly to the case of the price fluctuations [57], relevant information can be extracted from the statistics of the large values of the trading volume. Remembering the assertion that this quantity can act as a proxy for the flow of information, we can look at a extreme-value analysis of the trading volume as form of understanding to what extent the emergence of large values of that financial quantity (large information flow) for a given company affects the volume of trading of the remaining equities. In the present case, we define constrained correlation matrices to the percentile p, C_p_(*t*; *s*) where we consider that the contribution to the calculation of the entry C_p_(*t*; *s*)_*ij*_ does only count when the trading volume of one of the companies—either *i* or *j*—is in the top p% of trading volume values for that time *t* and semester *s*.

**Fig 8:**
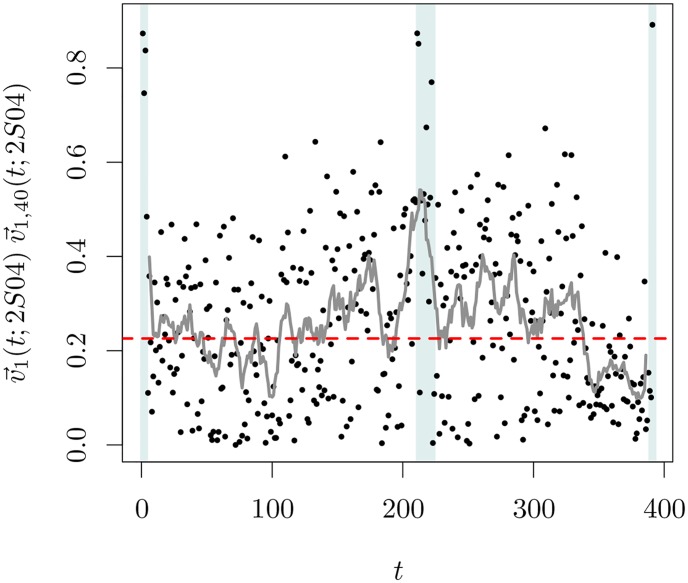
Overlap of the mode versor of the full correlation and the constrained correlation matrix in 2S04 v intraday time. The regions shadowed in grey represent the timestamp intervals with striking overlap between both versors that would correspond to angle less than 30 degrees between them. In this case, the average over intraday time (grey regions excluded) is equal to 0.26 ± 0.18 and the median equals 0.22. The solid grey line at time *t* corresponds to the local arithmetic average over a sample window starting at *t* − 5 and ending *t* + 5.

Because we are analysing the data on a 6-month basis—which implies a not so huge number of values for statistical purposes—we define p = 40. This means we effectively carry out a correlation analysis on the values above the median. In S3 Video, we show the evolution of C_40_(*t*; 2*S*04) across the trading session. The visual inspection of S3 Video hints at a rather modest level of similarity between the full and the constrained matrix for most of the time; nevertheless, the periods in which we have bursts in the λ_1_ are accompanied by large values of the constrained eigenvalue λ_1,40_. With the goal of quantitatively probing the differences and similarities between both matrices, we centre our attention on the modes of both matrices and compute the scalar product between the eigenversors of the largest eigenvalue of each case as presented in Fig 8. In that figure, the overlap between the two versors has a median across the trading session equal to 0.25. That overlap is quite strong—with values around 0.8—in the first minutes of the session and afterwards in the (effective) transition between the morning and the afternoon parts of the business day. Note that these are the periods where the kurtosis (individual and cross-sectional) reaches its largest values. Nevertheless, in this case, we cannot identify a statistically significant difference between the morning and the afternoon. Combining our observations we confirm that the emergence of large trading volumes lies at the basis of the collective trading dynamical behaviour, particularly in those two key periods of the business day.

### Nonstationary collective behaviour

#### Eigenversor analysis

**Fig 9:**
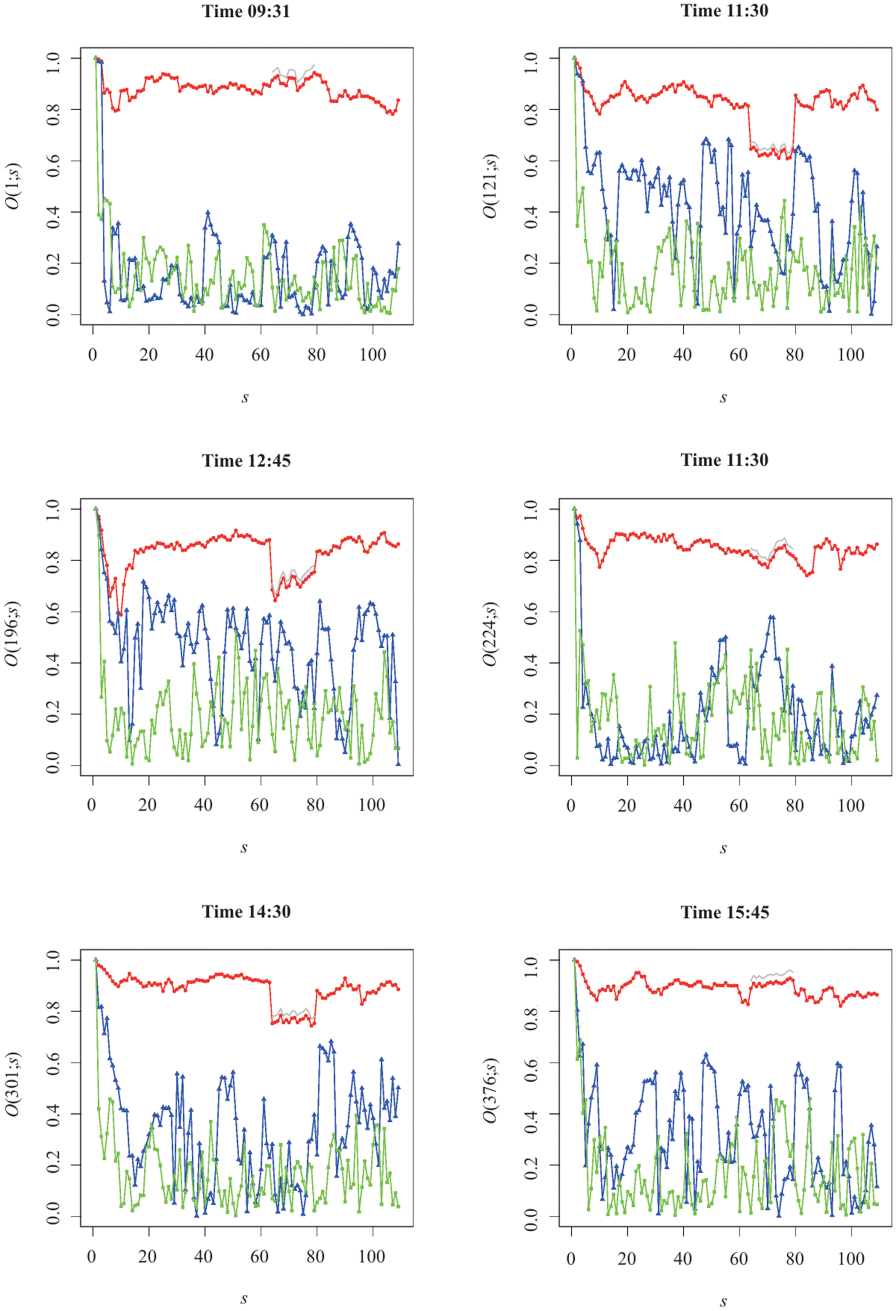
Overlap *O*_*α*_(*t*; *s*) for the three largest eigenversors v semester. The red, blue, green line+symbols represent the eigenversors of the first, second and third eigenvalues, respectively. The grey line corresponds to the value of the overlap adjusted by a factor 30/29.

As our previous study on the individual statistical properties showed, intraday features of the trading volume evolve in the long-term, namely the silhouette of the famous ∪-shape, which has been loosing concavity. In the case of the eigenversors, we can also try to understand how the weight in the eigenversors is reassigned as months go by. In order to do so, we analyse the evolution of the overlap

$$\begin{array}{c} O_{\alpha }\left(t;s\right)\equiv {\vec{v}}_{\alpha }\left(t;1\right)\cdot {\vec{v}}_{\alpha }\left(t;s\right) \end{array}$$ (21)

for different timestamps, as depicted in Fig 9.

From the panels therein, we observe that the first largest eigenversor was pretty coherent through the years 2003–2014, both in respect of the value in itself, with an average value of 0.84 ± 0.09, as well as the (small) fluctuations from one semester to the next. Yet, as Fig 9 indicates, for the timestamps at which we have peaks in λ_1_(*t*; *s*), there was a decrease in the value of the scalar product during the trading halt of General Motors stocks due to the bailout by the US Government. Interestingly, for a fixed intraday time *t*, if we compute the ratio between the average of *O*_*α*_(*t*; *s*) wherein GM.NY was traded and not traded we obtain a value *circa* 1.15, which is appreciably larger than 30/29 ≃ 1.03. The difference between the two ratios suggests General Motors plays a particular role in the collective dynamics of the trading volume of the this group of stocks and ultimately might help understand the decision to bail out the company.

For other eigenorders, eg, *α* = {2, 3}, the computation of *O*_*α*_(*t*; *s*) can be challenged because we have verified they are effectively within the Marchenko-Pastur limits and hence do not correspond to structured market modes, except for λ_2_ when the market opens and during the morning-afternoon transition. For that reason, we understand that: *i)* *O*_*α*_(*t*; *s*) fluctuates significantly and within noise level during morning/afternoon periods for which λ_1_ is close to its morning/afternoon mean value (within error) and *ii)*, for the timestamps where the burst in the value of the trading volume mode emerge (see Fig 3), we glimpse a slowly decaying trend for *O*_2_(*t*; *s*) whereas for *O*_3_(*t*; *s*) we reach noise level in the scale of a few months. This suggests that at these specific times the second largest eigenvalue can be relevant. Curiously, the last minute of the trading session is characterised by noise for all the eigenorders we have analysed.

#### Eigenvalue analysis

As pointed in the previous subsection, the intraday analysis of the correlation matrix of the trading volume has allowed us to identify a clear mode in the collective behaviour of the trading volume, which performs a 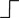-like profile across the day. With the goal of understanding how the intraday profile of the largest eigenvalue changed, we assume that each minute corresponds to a given dimension and transform the intraday profile into a versor,

$$\begin{array}{c} {\vec{\Lambda }}_{\alpha }\left(s\right)=\frac{1}{\sqrt{\sum_{t=1}^{390}\lambda_{\alpha }{\left(t;s\right)}^{2}}}\;[\lambda_{\alpha }\left(1;s\right){\hat{z}}_{1}+\lambda_{\alpha }\left(2;s\right){\hat{z}}_{2}+\ldots +\lambda_{\alpha }\left(390;s\right){\hat{z}}_{390}]. \end{array}$$ (22)

**Fig 10:**
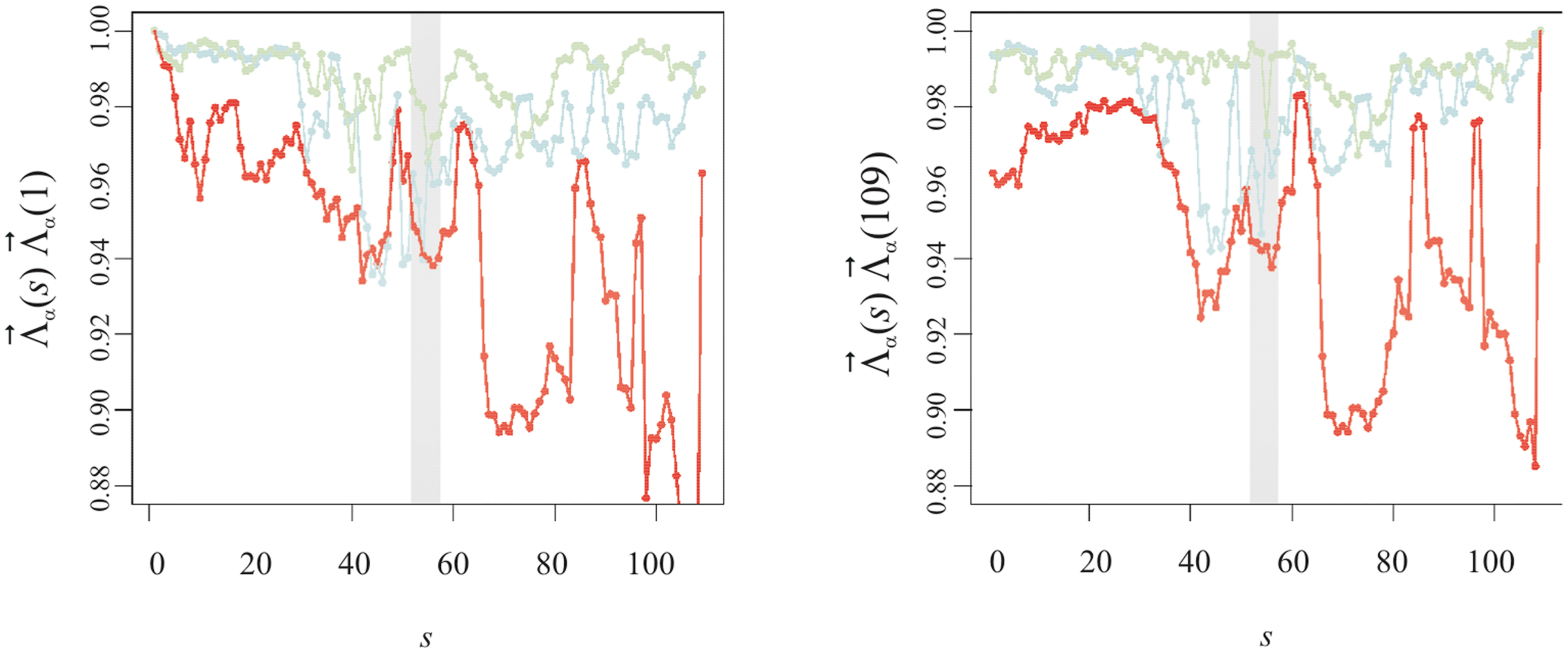
Overlap of the eigenvalue versor ${\vec{\Lambda }}_{\alpha }\left(s\right)$ with ${\vec{\Lambda }}_{\alpha }\left(1\right)$ v semester s (left panel) and ${\vec{\Lambda }}_{\alpha }\left(s\right)$ with ${\vec{\Lambda }}_{\alpha }\left(109\right)$ v semester s (right panel). The red curve represents the market mode versor (*α* = 1) and the pale blue and green lines are for *α* = 2, 3, respectively. The interval for which the background is grey defines the semesters containing at least one month from 2S08. The dashed horizontal lines in the insets represent the standard deviation of ${\vec{\Lambda }}_{\alpha }\left(s\right)-{\vec{\Lambda }}_{\alpha }\left(s-1\right)$ before and after 2S8. Their values are: 0.0049 and 0.015 on the left hand side inset and on the right hand side inset 0.019 and 0.0036.

We then compute the overlap between each ${\vec{\Lambda }}_{\alpha }\left(s\right)$ and ${\vec{\Lambda }}_{\alpha }\left(1\right)$. The outcome of these calculations, averaged over companies, is presented in Fig 10.

The overlap between the second and third largest eigenvalues profile-versors of semester *s* and semester 1 is more or less constant until semester 30 (2S06) whence it exhibits larger fluctuations. These overlap values are never less than 0.93 for *α* = 2 and 0.96 for *α* = 3. For the trading volume mode, we have a clear increase of the fluctuations from semester 58-off onwards. That is clear when we look into the fluctuations and apply a *F*-test of equivalence of the variance in two sub-groups. Accordingly, by separating those fluctuations into ‘before semester 58’ (the climax semester of the subprime crisis) and ‘after semester 58’ the statistical test allows us to assert that we have different behaviour for both groups with a significance of 95%. In order to appraise the robustness of this finding, we can also compute the fluctuations of the overlap with respect to other semesters—including the last one (shown on the right panel in Fig 10) obtaining equivalent results.

## Conclusion

With this work, we have expanded our previous study on individual and cross-sectional intraday features of trading volume. We have done so by analysing quantities derived from time-dependent correlation matrices of the trading volume of the DJIA components, namely its eigenvalues and eigenversors. By dividing the data into 6-month (overlapping) spells our study has assessed their nonstationary features in the last 10 years as well.

Employing Random Matrix Theory and Principal Component Analysis, we have computed at each trading minute, *t*, the spectrum of eigenvalues and verified the largest of them, λ_1_(*t*; *s*), is consistently off the upper bound imposed by the Marchenko-Pastur distribution of a random matrix under the same conditions. That proves the collective behaviour of the trading volume of the stocks composing the DJIA is ruled by a mode. Complementary, the remaining eigenvalues are within the limits established by random matrix theory for most of the time and hence we can associate them with noise. The exception is the second largest eigenvalue at the beginning of the trading session—wherein it can be viewed as a market mode for sure as well—and in the transition between the morning and afternoon part of the session for which its values are slightly above the mode limit. This finding establishes a clear and surprising difference between the collective behaviour of the trading volume and that of the price fluctuations: in spite of basically showing a white-noise autocorrelation function, the latter has a larger set of relevant eigenvalues of the correlation matrix [15]. Such properties contrast to those of the trading volume which exhibits a slowly decaying autocorrelation function, but only one statistically significant collective mode is systematically found. Furthermore, although λ_1_/*N* is larger for price fluctuations, the difference in the number of significant eigenvalues is even more surprising when we understand that, eg, λ_2_/*N* is equal to 0.07 for the trading volume and 0.02 for price fluctuations. At first, we have assumed that the different sample rating was at the helm of the somewhat conflicting results; however, a subsequent analysis has shown that despite the larger the lag, the larger the weight of the trading volume mode in the eigenspectrum, the other largest eigenvalues remain around or below the limits imposed by random matrix theory (Ignoring the market mode of the second largest value character in the very first minutes of the session.). In other words, when we bring into play the effects of asynchrony in trading to the analysis of the collective behaviour of the trading volume we fundamentally strengthen the collective dynamics in a linear first-order way. The absence of significant changes in second and third largest eigenvalues might be related to the ‘blue chip’ (high-liquidity) character of DJIA stocks and that second-order effects become more clear as we consider less capitalised (ergo less liquid) companies. We expect to investigate these observations in future work of ours.

Across the session, λ_1_(*t*; *s*) roughly defines a step-like shape punctuated with bursts in the very first minutes of the session and in the beginning of the afternoon part of the session, for which is not distinguishable a relaxation process. Applying standard statistical testing, we have confirmed that the trading volume is also collectively defined by two different regimes: before and after lunch. This feature bridges with the claims we asserted for a previous cross-sectional analysis: in the morning, the trading volume of the DJIA stocks are more loose and large trading volumes mainly stem from the impact of news disclosed in the overnight which have those informations passed on to the price as soon as the market opens; in the afternoon, the trading volume collective mode increases in the wake of a more concerted behaviour of the DJIA stocks so that the tails in the trading volume (cross-sectional) distribution are the outcome of large trading volumes among the companies composing the index.

The conclusions we have just conveyed are further supported by the analysis of the evolution of the scalar product between the first eigenversor and the uniform versor. The value of the projection of the former on the latter increases with intraday time *t* meaning that the companies tend to assume equivalent weights in the collective dynamics as the time elapses; moreover, the peaks in the kurtosis of the trading volume concur with plunges in that scalar product, ie, we depart from quasi-homogeneity with some company(ies) assuming some sort of leading role.

A subsequent analysis of the correlation matrix constrained to the larger values of the trading volume has proved that in the opening and after lunch—which are the periods for which we have the largest eigenvalue clearly beyond the Marchenko-Pastur limits —, the overlap (scalar product) between the first eigenversors of the full correlation matrix and the constrained correlation matrix reaches a value around 0.8, which indicates that the key element in the collective behaviour of the trading volume is the emergence of large values of *v*. Once again, we have been able to quantify the heuristic ‘lunch effect’, showing that agents do still take into account the start of the second half of the session in their trading strategies.

In a second stage, we have analysed how the quantities related to the correlation matrix of the trading volume have evolved across the last decade. Assuming the first semester of our data as default, we have tested the stationarity of both the first eigenversor and the intraday profile of the largest eigenvalue as well. Considering the results of the overlap with ${\vec{v}}_{1}\left(t;1\right)$, we have understood that ${\vec{v}}_{1}\left(t;s\right)$ is quite robust with typical values larger than 0.8. Importantly, we have grasped that for the timestamps corresponding to the peaks in λ_1_ there is a visible shifting down (of the overlap) in the semesters in which General Motors was not traded due to the Chapter 11 reorganisation process filled in June 2008. The ratio gap between the typical value of the overlap and that computed during the GM bailout trading halt is larger than 30/29, which points out to the relevance of that company in the market dynamics, especially in the spike intraday periods we have mentioned. It is worth recalling that GM was vehicle sales leader and the automotive sector is intimately related to several other economical sectors. For these timestamps, we have also noticed that the scalar product of ${\vec{v}}_{2}\left(t;s\right)$ by ${\vec{v}}_{2}\left(t;1\right)$ suggests that the overlap with the first semester endures for several months before attaining the noise level. On the other hand, with the goal of understanding the degree of robustness of the intraday profile of the eigenvalues, we have defined eigenvalue versors by assuming each minute of the trading session as an orthogonal dimension. The overlap of such versors, namely that composed of λ_1_(*t*; *s*), shows there is a definite change—supported by a *F*-test of statistical significance—in the behaviour of the overlap fluctuations before and after the second semester 2008 (it gets more flustered after that), the climax of the sub-prime crisis. This finding has been verified assuming other semesters as reference as well.

The present results—and those of the preceding Paper I—have shed light on the intraday dynamics of trading volume (of liquid stocks) in financial markets. Sustaining our analysis on the close relation between trading volume and information flow, we have been capable of explaining the reasons for the different statistical behaviour between the morning and the afternoon parts of the trading session. Nonetheless, several questions are still short of a quantitative approach besides liquidity matters; among them we can mention the intraday profile of the volatility and its relation to the trading volume from a correlation matrix point of view. Alternatively, bearing in mind the analysis carried out for the price fluctuations [58], it is also worthwhile to consider the cross behaviour of trading volume fluctuations [29] in order to get a better collective understanding of the changes in market activity—which have helped explain the power-law distribution of trading volume [20, 59, 60]—and the limits of acceptance of standard theories of diffusion of information in the market, namely the Mixture of Distributions Hypothesis and the alternative Sequential Arrival of Information Hypothesis.

## Supporting information

S1 Video Evolution of the correlation matrix C(*t*; 2*S*04) of the trading volume of the DJIA components for the times indicated in the title of each panel. Each point of the matrix is computed using Eq (7) and colour mapped according to the legend in the video.
(M4V)

S2 Video Evolution of the matrix C_S_(*t*; 2*S*04) of the trading volume of the DJIA components for the times indicated in the title of each panel. Each point of the matrix is computed using the absolute values of C(*t*; 2*S*04)—that are computed using Eq (7)—that are afterwards sorted along the rows in descending order and colour mapped according to the legend in the video.
(M4V)

S3 Video Evolution of the constrained matrix C_p_(*t*; 2*S*04) of the trading volume of the DJIA components for the times indicated in the title of each panel. Each entry of the matrix C_p_(*t*; *s*)_*ij*_ is computed considering only the pairs of values of *i* and *j* for which one of the companies—either *i* or *j*—is in the top 40% at time *t*.
(M4V)

## References

[1] Karpoff JM. The relation between price changes and trading volume: a survey. J Financ Quart Anal. 1987; 22:109 10.2307/2330874

[2] Clark PK. A Subordinated Stochastic Process Model with Finite Variance for Speculative Prices. Econometrica. 1973; 41:135 10.2307/1913889

[3] Sornette D. Critical phenomena in natural sciences: chaos, fractals, selforganization and disorder: concepts and tools. Berlin: Springer-Verlag; 2006.

[4] Epps TW and Epps ML. The Stochastic Dependence of Security Price Changes and Transaction Volumes: Implications for the Mixture-of-Distributions Hypothesis. Econometrica. 1976; 44:305 10.2307/1912726

[5] Tauchen G and Pitts M. The Price Variability-Volume Relationship on Speculative Markets. Econometrica. 1983; 51:485 10.2307/1912002

[6] Harris L. Transaction Data Tests of the Mixture of Distributions Hypothesis. J Finance Quant Anal. 1987; 22:127 10.2307/2330708

[7] Jones CM, Kaul G and Lipson ML. Transactions, volume, and volatility. Rev Finan Stud. 1994; 7:631 10.1093/rfs/7.4.631

[8] Engle RF, Focardi SM and Fabozzi FJ. ARCH/GARCH Models in Applied Financial Econometrics In: Fabozzi FJ, editor. Handbook of Finance, Volume III: Valuation, Financial Modeling, and Quantitative Tools. New York City—NY: John Wiley & Sons, New York; 2008.

[9] Lamoureux CG and Lastrapes W. Heteroscedasticity in stock returns data: volume versus GARCH effects. J Finance. 1990; 45:221 10.1111/j.1540-6261.1990.tb05088.x

[10] Copeland T. A Model of Asset Trading under the Assumption of Sequential Information Arrival. J Finance. 1976; 31:1149 10.1111/j.1540-6261.1976.tb01966.x

[11] Graczyk MB, Duarte Queirós SM. Intraday seasonalities and nonstationarity of trading volume in financial markets: individual and sectional features. PLoS ONE 11 2016; (11): e0165057 10.1371/journal.pone.0165057 27812141 PMC5094667

[12] Admati A, Pfleiderer P. A theory of intraday patterns: volume and price variability. Rev Financ Stud. 1988; 1:3 10.1093/rfs/1.1.3

[13] Jain PC, Joh G-H. The dependence between hourly prices and trading volume. J Finan Quant Anal. 1988; 23:269 10.2307/2331067

[14] Andersen T, Bollerslev T. Intraday periodicity and volatility persistence in financial markets. J Empir Financ. 1997; 4:115 10.1016/S0927-5398(97)00004-2

[15] Allez R, Bouchaud JP. Individual and collective stock dynamics: intra-day seasonalities. New J Phys. 2011; 13:025010 10.1088/1367-2630/13/2/025010

[16] Duarte Queirós SM. Trading volume in financial markets: An introductory review. Chaos Solitons Fractals. 2016; 88:24 10.1016/j.chaos.2015.12.024

[17] Osorio R, Borland L, Tsallis C. Distributions of high-frequency stock-market observables In: Gell-Mann M, Tsallis C, editors. Nonextensive Entropy: Interdisciplinary Applications. New York City—NY: Oxford University Press; 2004.

[18] de Souza J, Moyano LG and Duarte Queirós SM. On statistical properties of traded volume in financial markets. Eur Phys J B. 2006; 50:165 10.1140/epjb/e2006-00130-1

[19] Mu G-H, Chen W, Kertész J, Zhou W-X. Preferred numbers and the distributions of trade sizes and trading volumes in the Chinese stock market. Eur Phys J B. 2009; 68:245 10.1140/epjb/e2009-00059-9

[20] Gopikrishnan P, Plerou V, Gabaix X, Stanley HE. Statistical properties of share volume traded in financial markets. 2000; Phys Rev E 62:R4493 10.1103/PhysRevE.62.R4493 11089066

[21] Duarte Queirós SM, Moyano LG. Yet on statistical properties of traded volume: Correlation and mutual information at different value magnitudes. Physica A. 2007; 383:10 10.1016/j.physa.2007.04.082

[22] Moyano LG, de Souza J and Duarte Queirós SM. On the multi-fractal structure of traded volume in financial markets. Physica A. 2006; 371:118 10.1016/j.physa.2006.04.098

[23] Li W, Wang F, Havlin S and Stanley HE. Financial Factor Influence on Scaling and Memory of Trading Volume in Stock Market. Phys Rev E. 2011; 84:046112 10.1103/PhysRevE.84.046112 22181232

[24] Eisler Z and Kértesz J. Scaling theory of temporal correlations and size-dependent fluctuations in the traded value of stocks. Phys Rev E. 2006; 73:046109 10.1103/PhysRevE.73.046109 16711880

[25] Eisler Z and Kértesz J. Size matters: some stylized facts of the stock market revisited. Eur Phys J B. 2006; 51:145 10.1140/epjb/e2006-00189-6

[26] Eisler Z and Kértesz J. Liquidity and the multiscaling properties of the volume traded on the stock market. Europhys Lett. 2007; 77:28001 10.1209/0295-5075/77/28001

[27] Plerou V and Stanley HE. Tests of Scaling and Universality of the Distributions of Trade Size and Share Volume: Evidence from Three Distinct Markets. Phys Rev E. 2007; 76:046109 10.1103/PhysRevE.76.046109 17995062

[28] Rocha P, Raischel F, Boto JP and Lind PG. Optimal models of extreme volume-prices are time-dependent. J Physics: Conf Series. 2015; 574:012148.

[29] Podobnik B, Horvatic D, Petersen AM and Stanley HE. Cross-correlations between volume change and price change. Proc Nat Acad Sci USA. 2009; 106:22079 10.1073/pnas.0911983106 20018772 PMC2799689

[30] Wigner EP. On a class of analytical functions from the quantum theory of collisions. Ann Math. 1951; 53:36 10.2307/1969342

[31] Wigner EP. On the statistical distribution of the widths and spacings of nuclear resonance levels. Proc Cambridge Philos Soc. 1951; 47:479 10.1017/S0305004100027237

[32] Wigner EP. Results and theory of resonance absorption. In: Block RC, Good WM, Harvey JA, Schmitt HW and Trammell GT, editors. Conference on Neutron Physics by Time-of-Flight held at Gatlinburg—TE November 1 and 2 1956. Oak Ridge—TE: Oak Ridge National Laboratory.; 1956.

[33] Mezard, Parisi G and Virasoro M. Spin glass theory and beyond. Singapore: World Scientific;1986.

[34] Bohigas O, Giannoni MJ, Schmit S. Characterization of Chaotic Quantum Spectra and Universality of Level Fluctuation Laws. Phys Rev Lett. 1984; 52:1 10.1103/PhysRevLett.52.1

[35] Bahcall SR. Random Matrix Model for Superconductors in a Magnetic Field. Phys Rev Lett. 1996; 77:5276 10.1103/PhysRevLett.77.5276 10062760

[36] Beenakker CWJ. Random-matrix theory of quantum transport. Rev Mod Phys. 1997; 69:731 10.1103/RevModPhys.69.731 10054300

[37] Laird NM and Ware JH. Random-Effects Models for Longitudinal Data. Biometrics. 1982; 38:963 10.2307/2529876 7168798

[38] Rue H and Held L. Gaussian Markov Random Fields: Theory and Applications. Boca Raton—FL: CRC Press; 2005.

[39] Huang G-B, Zhu Q-Y and Siew C-K. Extreme learning machine: Theory and applications. Neurocomputing. 2006; 70:489 10.1016/j.neucom.2005.12.126

[40] Kritchamn Sh and Nadler B. Non-Parametric Detection of the Number of Signals: Hypothesis Testing and Random Matrix Theory. IEEE Trans Sig Process. 2009; 57:3930; 10.1109/TSP.2009.2022897

[41] Titman S and Wessels R. The Determinants of Capital Structure Choice. 1988; J Financ 43:1 10.1111/j.1540-6261.1988.tb02585.x

[42] Bouchaud JP, Potters M. An Introduction to Econophysics: Correlations and Complexity in Finance. Cambridge: Cambridge University Press; 2000.

[43] Laloux L, Cizeau P, Potters M and Bouchaud JP. Random matrix theory and financial correlations. Int J Theor Appl Finan. 2000; 3:391 10.1142/S0219024900000255

[44] Bouchaud JP and Potters M. In: Akemann, Baik J and Di Francesco, editors. The Oxford Handbook on Random Matrix Theory. Oxford: Oxford University Press; 2011.

[45] Plerou V, Gopikrishnan P, Rosenow B, Amaral LAN, and Stanley HE. Universal and Nonuniversal Properties of Cross Correlations in Financial Time Series. Phys Rev Lett. 1999; 83:1471 10.1103/PhysRevLett.83.1471

[46] Mantegna RN. Hierarchical structure in financial markets. Eur Phys J B. 1999; 11:193 10.1007/s100510050929

[47] Mantegna RN, Stanley HE. An Introduction to Econophysics: Correlations and Complexity in Finance. Cambridge: Cambridge University Press; 1999.

[48] Engle R. Dynamic Conditional Correlation: A Simple Class of Multivariate Generalized Autoregressive Conditional Heteroskedasticity Models. J Bus Econ Stat. 2002; 20:339 10.1198/073500102288618487

[49] Markowitz HM. Harry Markowitz: Selected Works. Hackensack—NJ: World Scientific; 2009.

[50] Rebonato R and Jäckel P. The Most General Methodology to Create a Valid Correlation Matrix for Risk Management and Option Pricing Purposes.; 2011. Available at SSRN: http://ssrn.com/abstract=1969689

[51] Baik J, Ben Arous G and Péché S. Phase transition of the largest eigenvalue for non-null complex sample covariance matrices. Ann Prob. 2005; 33:1643 10.1214/009117905000000233

[52] Camargo S, Duarte Queirós SM and Anteneodo C. Bridging stylized facts in finance and data nonstationarities. Eur Phys J B. 2014; 86:159 10.1140/epjb/e2013-30974-9

[53] Gopikrishnan P, Plerou V, Gabaix X and Stanley HE. Statistical properties of share volume traded in financial markets. Phys Rev E. 2000; 62:R4493 10.1103/PhysRevE.62.R4493 11089066

[54] Duarte Queirós SM and Moyano LG. Yet on statistical properties of traded volume: Correlation and mutual information at different value magnitudes. Physica A. 2007; 383:10 10.1016/j.physa.2007.04.082

[55] Borghesi C, Marsili M and Miccichè S. Emergence of time-horizon invariant correlation structure in financial returns by subtraction of the market mode. Phys Rev E. 2007; 76:026104 10.1103/PhysRevE.76.026104 17930101

[56] Epps TW. Comovements in Stock Prices in the Very Short Run, J Am Stat Assoc. 1979; 74:291.

[57] Embrechts P, Klüppelberg C and Mikosch T. Modelling Extremal Events for Insurance and Finance. Berlin: Springer; 1997.

[58] Duarte Queirós SM and Graczyk MB. Nonstationarity of the intraday individual and collective seasonalities of price fluctuations. Journal of Network Theory in Finance.; 2017 10.21314/JNTF.2017.026

[59] Lux T and Marchesi M. Scaling and criticality in a stochastic multi-agent model of a financial market. Nature. 1999; 397:498 10.1038/17290

[60] Duarte Queirós SM. On the distribution of high-frequency stock market traded volume: a dynamical scenario. Europhys Lett. 2005; 71:339.

